# Hypocalcemia and Hypophosphatemia following Concurrent Denosumab Injection and Ferric Carboxymaltose Infusion in a Patient with Normal Renal Function

**DOI:** 10.1155/2024/8910092

**Published:** 2024-02-01

**Authors:** Naomi Szwarcbard, Chloe Dawson, Lai-Ming Kathleen Pak, Kathryn L. Hackman

**Affiliations:** ^1^Department of Endocrinology & Diabetes, Alfred Hospital, Melbourne, Australia; ^2^Department of Medicine, Monash University, Melbourne, Australia

## Abstract

Hypocalcemia following denosumab administration is well described. Hypophosphatemia following an intravenous iron infusion is an increasingly recognized adverse effect. Intravenous iron preparations increase fibroblast growth factor 23 (FGF23) levels. This both stimulates renal phosphate excretion and reduces 1,25-dihydroxyvitamin D (1,25(OH)_2_D) levels, resulting in reduced calcium absorption. Both osteoporosis and iron deficiency are common and frequently co-occur. The convenience and efficacy of both denosumab, a subcutaneous injection, and ferric carboxymaltose (Ferinject®), a 15-minute intravenous infusion, both of which can be given in the primary care setting, make these preferred treatment options. However, prescribers are often unaware of potential adverse outcomes, especially when these medications are given in tandem. We present a case of symptomatic hypocalcemia and hypophosphatemia in a 29-year-old woman with myelin oligodendrocyte glycoprotein antibody-associated disease (MOGAD) and normal renal function, in the setting of concurrent denosumab and ferric carboxymaltose administration for treatment of glucocorticoid-induced osteoporosis and iron deficiency anemia.

## 1. Introduction

Iron deficiency, with or without resultant anemia, can occur in the setting of poor intake or absorption of dietary iron, chronic kidney disease (CKD), heavy blood loss, or acute or chronic infection. It is the leading cause of anemia worldwide and is associated with increased morbidity and mortality, as well as significant global health and economic consequences [1]. Intravenous iron infusions provide a safe and effective option to treat iron deficiency when oral iron preparations are either poorly absorbed or result in significant gastrointestinal symptoms [2], and their prescription has significantly increased over recent years [3]. A rare but increasingly well recognized complication of intravenous iron is hypophosphatemia. This is precipitated by FGF23-driven renal phosphate wasting and is commonest following infusion with the ferric carboxymaltose iron preparation [4, 5]. Increased FGF23 following intravenous iron also reduces hydroxylation of 25-hydroxyvitamin D to 1,25(OH)_2_D levels, with the potential to cause hypocalcemia, via reduced gastrointestinal absorption, though usually to a milder degree compared with phosphate losses [6–8]. Hypocalcemia following denosumab administration is a well-known side effect that can be severe, especially in the setting of CKD or vitamin D deficiency [9].

Intravenous iron and subcutaneous denosumab both have the potential to interrupt calcium and phosphate homeostasis. These adverse effects may be exacerbated by coadministration. Given the increasing prescription of both denosumab and intravenous iron, clinicians need to be aware of the potential for adverse effects, avoid concurrent administration when possible, and monitor for symptoms of biochemical derangement.

## 2. Case Presentation

A 29-year-old woman had been diagnosed with myelin oligodendrocyte glycoprotein antibody-associated disease (MOGAD), complicated by recurrent episodes of optic neuritis and an atonic bladder. She had required immunosuppression regimens since diagnosis in 2016, initially with mycophenolate mofetil and azathioprine, followed by a combination of rituximab, methotrexate, and prednisolone. In the setting of ongoing flares of optic neuritis, the rituximab infusions were ceased, and she commenced both tocilizumab and plasma exchange therapy.

Due to her risk of glucocorticoid-induced osteoporosis, she was treated with oral alendronate from 2017 to 2020 and then switched to 6-monthly denosumab in 2021. Two days following her last 60 mg/ml denosumab injection, she received a 1000 mg intravenous ferric carboxymaltose iron infusion for iron deficiency anemia. Over the next two weeks, the patient reported worsening perioral and peripheral parasthesia, muscle spasms, and increasing fatigue, resulting in a presentation to the emergency department of our hospital for further assessment. Given the relatively rapid time course of electrolyte derangement and supplementation, there were no associated skeletal features such as bone pain or altered gait.

Baseline pathology (see Table 1) performed the day prior to the denosumab administration demonstrated normal biochemistry: corrected calcium 2.23 mmol/L (2.15–2.65 mmol/L), phosphate 0.85 mmol/L (0.75–1.5 mmol/L), and magnesium 0.85 mmol/L (0.70–1.10 mmol/L). She had evidence of iron deficiency anemia: hemoglobin 104 g/L (113–159 g/L), mean cell volume 83 fL (80–97 fL), and ferritin 8 mcg/L (20–300 mcg/L). Her renal function was normal (eGFR >90 ml/min/1.73 m^2^), 25 (OH) vitamin D was replete at 58 nmol/L (>50 nmol/L), and her alkaline phosphatase (ALP) level was low normal at 31 units/L (30–110 units/L).

On presentation to the emergency department two weeks postdenosumab and ferric carboxymaltose administration, she had significant biochemical derangements (see Table 1): corrected calcium 1.89 mmol/L, ionized calcium 1.04 mmol/L (1.15–1.3 mmol/L), and an undetectable phosphate (<0.23 mmol/L). Magnesium was normal at 0.91 mmol/L. There was improvement in her anemia with a hemoglobin of 123 g/L and mean cell volume of 93 fL, and the ALP level remained unchanged at 30 units/L.

In the emergency department, the patient initially received 20 mmol of intravenous potassium dihydrogen phosphate replacement. She was discharged home later that day on oral electrolyte replacements, requiring a maximum dose of sodium phosphate monobasic (4000 mg BD), calcium carbonate (1200 mg BD), and calcitriol (0.25 mcg BD). She reported symptom resolution within one day of electrolyte replacement, but experienced significant diarrhea associated with the high-dose phosphate replacement.

The patient's electrolyte replacements were weaned over a two-month period and then ceased, with maintenance of normal electrolyte levels. She was advised to avoid ferric carboxymaltose infusions in the future.

## 3. Discussion

This case highlights the risks of the concurrent use of denosumab and ferric carboxymaltose, which resulted in an unplanned emergency department presentation with symptomatic hypocalcemia and hypophosphatemia for this patient. The potential for similar clinical outcomes requires increased awareness among prescribing clinicians.

Denosumab is a monoclonal antibody that blocks the binding of the receptor activator of nuclear factor kappa-B ligand (RANKL) to the receptor activator of nuclear factor kappa-B (RANK). Blocking this binding prevents the usual pathway that signals for proliferation, maturation, and activation of osteoclasts. Hypocalcemia post-denosumab is a well-described complication, to the extent that denosumab has been used in the acute management of hypercalcemia [9]. CKD and the disordered bone metabolism that accompanies it are considered significant risk factors for denosumab-induced hypocalcemia due to a “hungry bone syndrome” phenomenon that reduces calcium release from bone [10].

Intravenous iron infusions are associated with hypophosphatemia, though their incidence varies depending on the preparation of the intravenous iron. In a recent meta-analysis including 42 clinical trials across a 15-year period, the incidence of hypophosphatemia following ferric carboxymaltose infusion was as high as 47% compared with 4% of patients infused with ferric derisomaltose, yet clinical awareness of this phenomenon remains variable [4].

**Figure 1:**
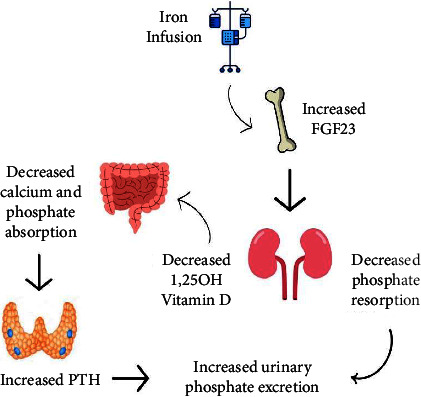
Mechanism of intravenous iron-induced hypophosphatemia.

Hypophosphatemia is mediated by an elevation in FGF23; a protein hormone secreted primarily by osteoblasts and osteocytes in response to elevated phosphate levels. Iron deficiency anemia itself results in increased FGF23 levels; however, due to a concurrent increase in FGF23 cleavage, there is no net effect on phosphate levels. It is postulated that ferric carboxymaltose iron preparations result in blocked cleavage and therefore reduced breakdown of FGF23, resulting in an overall increase in FGF23 levels. FGF23, bound to its transmembrane protein coreceptor Klotho, signals to reduce renal proximal tubule phosphate reabsorption via the sodium phosphate cotransporter 2a (NaPi2a) channel, driving increased renal phosphate losses. FGF23 also suppresses 1*α*-hydroxylase activity, resulting in decreased 1,25(OH)_2_D levels, leading to reduced intestinal phosphate and calcium absorption, and a compensatory secondary hyperparathyroidism (see Figure 1) [6–8, 11]. Compensatory hyperparathyroidism, aimed at preventing severe hypocalcemia in this context, results in further prolongation of renal phosphate wasting, even after FGF23 levels have normalized [6].

Phosphate is an essential component of our metabolism and forms a key requirement for bone mineralization, phospholipid cell membranes, nucleic acids, and phosphorylated enzymes [7]. Hypophosphatemia following an iron infusion may be mild and asymptomatic or severe, resulting in generalized muscle and bone pain, weakness, fatigue, nausea, and dizziness. There have been case reports of osteomalacia and fractures secondary to iron infusion-induced hypophosphatemia [4, 6, 12]. Risk factors for iron infusion-induced hypophosphatemia include preserved renal function, low baseline phosphate and ferritin levels, vitamin D deficiency, and pre-existing hyperparathyroidism [4, 12]. The ferric carboxymaltose iron preparation is associated with the highest risk of hypophosphatemia. The risk of hypophosphatemia also increases with the number of iron infusions administered [4–6]. The nadir phosphate level is likely to be reached within two to three weeks following an iron infusion, and although usually self-limiting and transient, hypophosphatemia occasionally persists beyond 6 months [5].

Treatment of iron infusion-induced hypophosphatemia is guided by the severity of the clinical presentation and the time course of persisting deficiencies. Treatment involves a combination of calcitriol (active vitamin D) and phosphate replacement, as well as calcium replacement if there is concurrent hypocalcemia. Patients with a vitamin D deficiency should also receive cholecalciferol. Additional treatment strategies aimed at preventing further episodes of hypophosphatemia include management of the underlying cause of iron deficiency, as well as the use of an alternative intravenous iron preparation if further infusions are required [4, 6].

To date, there has been one prior case report describing hypocalcemia and hypophosphatemia following concurrent denosumab and intravenous iron (ferric carboxymaltose) use in a patient with normal renal function [13]. It has also been reported in a patient with CKD, where background abnormal bone metabolism plays a significant role [10]. In these patients, denosumab-induced hypocalcemia is caused by net calcium influx into the bone, analogous to “hungry bone syndrome,” and impaired release of compensatory PTH due to FGF23 elevation attributable to CKD.

In patients with normal renal function, denosumab also inhibits osteoclast activation and differentiation, preventing bone resorption and thereby reducing calcium and phosphorous efflux from bone. Increased FGF23 from intravenous iron lowers 1,25(OH)_2_D levels and reduces compensatory PTH release, promoting hypocalcemia and hypophosphatemia due to reduced gastrointestinal absorption [10, 13, 14]. This combination of reduced gastrointestinal absorption and bone resorption of both calcium and phosphate, with concurrent renal phosphate wasting, as a result of coadministration of denosumab and intravenous iron, resulted in the emergency presentation of our patient. This highlights that the combination of these medications may disrupt multiple homeostatic pathways, with the potential to cause severe and prolonged electrolyte derangement [10, 13].

Despite the high incidence of hypophosphatemia following iron infusions, this phenomenon remains under-appreciated and relatively poorly understood. Its coadministration with denosumab may further exacerbate both hypophosphatemia and hypocalcemia. The increasing use of intravenous iron infusions has the potential to result in significant numbers of patients presenting with symptomatic electrolyte derangements. Given this potential impact, modifiable risk factors, such as pre-existing hypocalcemia, hypophosphatemia, and vitamin D deficiency, should be screened for and corrected prior to administration, and coadministration of intravenous iron and denosumab should be avoided.

## Figures and Tables

**Table 1:** Pathology results at baseline and on presentation to the emergency department following denosumab and ferric carboxymaltose administration.

	Baseline pathology	Emergency department pathology
Corrected calcium (2.15–2.65 mmol/L)	2.23 mmol/L	1.89 mmol/L
Ionized calcium (1.15–1.30 mmol/L)	—	1.04 mmol/L
Phosphate (0.75–1.5 mmol/L)	0.85 mmol/L	<0.23 mmol/L
Magnesium (0.70–1.10 mmol/L)	0.85 mmol/L	0.91 mmol/L
Estimated glomerular filtration rate (eGFR) (>90 ml/min/1.73 m^2^)	>90 ml/min/1.73 m^2^	>90 ml/min/1.73 m^2^
Vitamin D (>50 nmol/L)	58 nmol/L	—
Alkaline phosphatase (30–110 units/L)	31 units/L	30 units/L
Hemaglobin (113–159 g/L)	104 g/L	123 g/L
Mean cell volume (MCV) (80–97 fL)	83 fL	93 fL
Ferritin (20–300 mcg/L)	8 mcg/L	—

## Additional Points

*Learning Points*. (i) Intravenous iron increases FGF23, likely via impaired degradation, resulting in renal phosphate wasting from the proximal tubule and hypophosphatemia. (ii) Hypophosphatemia may be asymptomatic or result in generalized symptoms such as fatigue, muscle pain and weakness, nausea, and dizziness. There are also case reports of osteomalacia and fractures. (iii) Risk factors for iron-induced hypophosphatemia include pre-existing low levels of calcium, phosphate, ferritin, and vitamin D; normal renal function; use of the ferric carboxymaltose iron preparation; and the administration of multiple iron infusions. (iv) FGF23 also reduces 1*α*-hydroxylase activity. This reduces 1,25-dihydroxyvitamin D levels which may result in hypocalcemia via reduced intestinal calcium absorption. (v) Hypocalcemia is not commonly seen when iron infusions are administered alone due to compensatory hyperparathyroidism, but is more likely in the setting of denosumab use, when calcium efflux from bone is limited. (vi) Concurrent administration of denosumab and intravenous iron should be avoided as it has the potential to result in significant disruption to calcium and phosphate homeostasis.
